# MASLD or MetALD? Unveiling the Role of Alcohol in Liver Disease Progression in Diabetic Patients

**DOI:** 10.3390/biomedicines14010082

**Published:** 2025-12-31

**Authors:** Ermina Stratina, Carol Stanciu, Robert Nastasa, Sebastian Zenovia, Remus Stafie, Adrian Rotaru, Stefan Chiriac, Irina Girleanu, Cristina Muzica, Horia Minea, Laura Huiban, Anca Trifan

**Affiliations:** 1 Department of Gastroenterology, Grigore T. Popa University of Medicine and Pharmacy, 700115 Iasi, Romania; stratina.ermina@yahoo.com (E.S.); stanciucarol@yahoo.com (C.S.); sebastianzenovia20@gmail.com (S.Z.); stafieremus@gmail.com (R.S.); adrianrotaru94@yahoo.com (A.R.); stefannchiriac@yahoo.com (S.C.); gilda_iri25@yahoo.com (I.G.); lungu.christina@yahoo.com (C.M.); horia.minea@yahoo.com (H.M.); huiban.laura@yahoo.com (L.H.); ancatrifan@yahoo.com (A.T.); 2 Institute of Gastroenterology and Hepatology, “St. Spiridon” Emergency Hospital, 700111 Iasi, Romania

**Keywords:** metabolic dysfunction-associated steatotic liver disease, metabolic dysfunction and alcohol-related disease, transient elastography, type 2 diabetes mellitus, liver disease progression

## Abstract

**Background**: The transition from the term non-alcoholic fatty liver disease (NAFLD) to steatotic liver disease (SLD), an umbrella term for several related conditions, offers benefits, particularly in identifying cardiometabolic risk factors more effectively. However, the impact of alcohol consumption on liver disease progression remains significant, leading to the recognition of a new entity: MetALD (metabolic dysfunction-associated steatotic liver disease with moderate alcohol intake). **Aim**: This study aimed to compare characteristics associated with liver disease progression in diabetic patients diagnosed with metabolic dysfunction-associated steatotic liver disease (MASLD) versus those with MetALD. **Materials and Methods**: In this prospective study, 286 diabetic patients were followed for 12 months. All patients underwent transient elastography (TE) and ultrasound to assess hepatic steatosis. Participants were classified into MASLD and MetALD groups. The performance of fibrosis-4 index (FIB-4), and NAFLD fibrosis score (NFS) were also evaluated. **Results**: MASLD was diagnosed in 58.2% (167 patients), of whom 4.9% (7 patients) had TE values suggestive for liver cirrhosis. Among those with MetALD, 17.6% (21 patients) had TE values compatible with advanced fibrosis. MASLD subjects presented a slight decrease in liver fibrosis values from 6.58 ± 2.27 kPa to 6.03 ± 1.57 kPa in the 12 months. On the contrary, MetALD subjects had an increase of liver stiffness measurements (LSM) values from 11.83 ± 6.27 kPa to 12.24 ± 8.66 kPa. **Conclusions**: in diabetic patients, the coexistence of moderate alcohol intake and cardiometabolic risk factors (MetALD) is associated with more advanced liver fibrosis and impaired long-term glycemic control, compared to MASLD alone.

## 1. Introduction

A unified global approach to the nomenclature and definition of steatotic liver disease (SLD) is essential to increase awareness of the disease, change policies, identify individuals at risk, and facilitate diagnosis and access to care. It has always been felt that the term “non-alcoholic” did not accurately capture the etiology of the disease, and, in particular, the term “fatty” was stigmatizing by some. In addition, there are individuals with risk factors for non-alcoholic fatty liver disease (NAFLD), such as type 2 diabetes mellitus (T2DM), who consume more alcohol than the relatively strict limits used to define the non-alcoholic nature of the disease, who were not adequately recognized by the previous nomenclature and were excluded from studies [1,2].

Thus, in June 2023, the new classification defined as metabolic dysfunction-associated steatosis liver disease (MASLD) emerged, encompassing liver steatosis in the presence of one or more cardiometabolic risk factors and the absence of harmful factors such as alcohol consumption. The spectrum of MASLD includes steatosis, metabolic dysfunction-associated steatohepatitis (MASH, formerly non-alcoholic steatohepatitis), cirrhosis, and hepatocarcinoma associated with MASH [3].

The group of metabolic factors established for the diagnosis of MASLD include abdominal obesity, hypertension, hyperglycemia, high triglyceride levels and low high-density lipoproteins (HDL) cholesterol levels [4]. Patients with liver steatosis who do not meet cardiometabolic criteria are included in the definition of cryptogenic SLD. MASLD and alcoholic liver disease (ALD) (alcohol consumption >50 g/day for women and >60 g/day for men) comprise the most common causes of SLD [5].

A new category, requiring further characterization, namely metabolic dysfunction-associated steatotic liver disease with moderate alcohol intake (MetALD), describes those with MASLD who consume higher amounts of alcohol (between 20 and 50 g/day for women and 30–60 g/day for men, respectively), but do not meet the criteria for ALD. Interestingly, despite sharing the same prevalence of cardiometabolic risk factors, without any clinical presentation difference from MASLD patients, MetALD is associated with a higher risk of all-cause mortality, and an increased risk of a hepatic decompensation event, supporting it as a distinct subclass of SLD with a more severe prognosis [6].

Prospective data does not adequately define the new SLD categories’ frequency and severity in Europe. Publications using the NHANES cohort showed that the prevalence of SLD varied from 34.6% to 42.15%, MASLD from 31.1% to 37.7%, and MetALD from 2% to 3.9%. Additionally, it is anticipated that the prevalence of advanced fibrosis ranges from 5.9% to 9.5% in people with MetALD and from 7.6% to 20.86% in patients with MASLD [7]. Common clinical metabolic diseases like T2DM, hypertension, and hyperlipidemia have also been demonstrated to be significant risk factors for the development of MASH, advanced fibrosis, and even severe liver disease outcomes like cirrhosis, decompensation, or liver-related death. When these conditions are combined with ethanol consumption, their negative hepatic impact is synergistic rather than additive, especially when genetic polymorphisms present, thus increasing the risk by eightfold of liver-related mortality [8].

Currently, there are two noninvasive ways to evaluate fibrosis: the first is a biological approach that makes use of aspartate aminotransferase (AST)-to-platelet ratio index and serum biomarkers of fibrosis, including the Fibrosis-4 Index (FIB-4), BARD score which is composed from BMI (Body Mass Index), AST/ALT ratio (Aspartate Aminotransferase to Alanine Aminotransferase ratio), and Diabetes, Forns, and NAFLD Score. According to Hargstrom et al., this approach has moderate diagnostic performance and an area under the curve (AUROC) of 0.54 to 0.71 [9]. The second approach is a physical technique that uses transient elastography (TE) with controlled attenuation parameter (CAP) (FibroScan^®^; Echosens, Paris, France) to quantify liver stiffness (LSM) [10]. When CAP is used, LSM has a high diagnostic accuracy for determining the degree of fibrosis and steatosis [11]. It is necessary to evaluate metabolic risk factors and alcohol consumption that meet the criteria for MetALD, as these patients tend to experience more rapid development of liver disease complications and have a worse prognosis compared to those with MASLD. The economic, social and public health burden determined by MASLD and MetALD imposes the need for comparative analysis of these patient groups [12].

This study will compare diabetic patients with MASLD and MetALD and examine how the severity of liver steatosis and fibrosis relates to metabolic disease progression risk.

## 2. Materials and Methods

### 2.1. Patients

The present research is a longitudinal prospective study carried out in the Institute of Gastroenterology and Hepatology within the County Emergency Clinical Hospital “St. Spiridon”, between May 2024 and May 2025, in which 286 diabetic patients who had no known chronic liver disease were included to evaluate the prevalence of liver steatosis, advanced fibrosis and cirrhosis due to MetALD, and MASLD. Also, the patients were evaluated in collaboration with the diabetes and nutritional diseases clinic within the same hospital.

Initially, the study participants received a standardized research visit that included a physical examination, alcohol questionnaires including Alcohol Use Disorder Identification Test (AUDIT) to establish the alcohol consumption, biological and immunological parameters from the laboratory, TE with CAP, and abdominal ultrasound evaluation. Therefore, 286 diabetic patients over the age of eighteen who have no history of chronic liver disease and who sign an informed consent form attest to their willingness to participate in the study meet the inclusion criteria. Also, they had to have a CAP value ≥ 274 dB/m using TE to show evidence of hepatic steatosis [10]. The definition of MASLD patients includes the presence of at least one cardiometabolic risk factor and alcohol consumption not exceeding 140 g per week for females and 210 g per week for males. Individuals with MetALD exhibited higher alcohol consumption, quantified as 140 to 350 g/week for females and 210 to 420 g/week for males [3,13].

**Figure 1:**
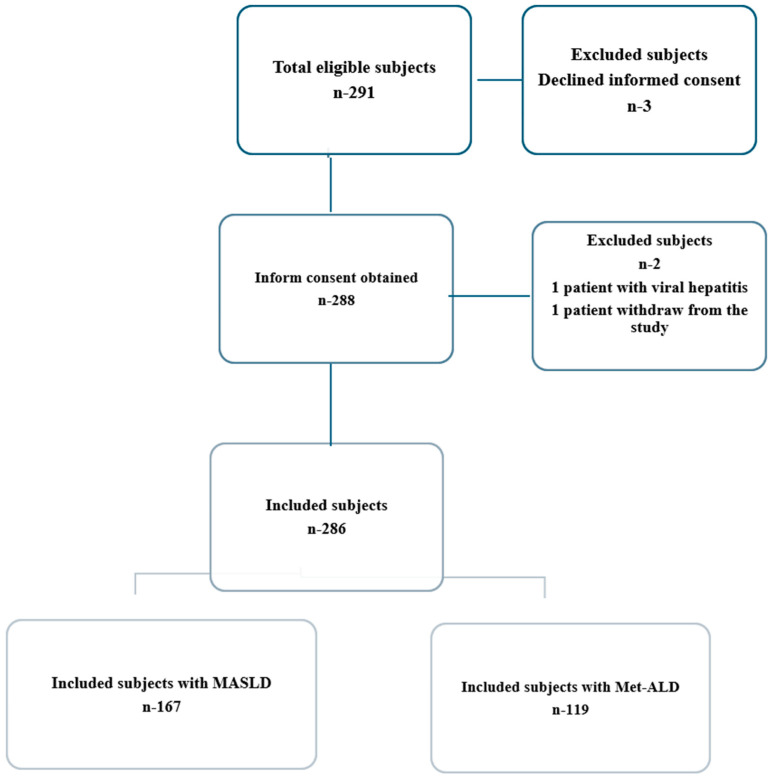
Study participant flow chart.

Patients with a history of chronic viral hepatitis infection or other causes of chronic liver disease (autoimmune hepatitis, Wilson disease, hemochromatosis, HIV co-infection, alcoholic liver disease), pregnancy, cardiac pacemakers, cancer, end-stage renal disease, life expectancy less than five years, ascites or hepatocellular carcinoma at ultrasound examination, aspartate aminotransferase (AST)/alanine aminotransferase (ALT) ≥ 5 times the upper limit of normal, or total bilirubin level ≥ 5 mg/dL, steatogenic medication (chemotherapy, corticosteroids, tamoxifen, methotrexate, amiodarone, estrogens) or an unreliable or unsuccessful TE examination were all excluded (Figure 1). The study was approved by our hospital’s Ethics Committee (approval number 411) and carried out in accordance with the principles of the Declaration of Helsinki. However, the inclusion criteria in the two categories, MASLD and Met-ALD, followed the Delphi consensus. The definition of MASLD patients includes the presence of at least one cardiometabolic risk factor and alcohol consumption not exceeding 140 g per week for females and 210 g per week for males. Individuals with MetALD exhibited higher alcohol consumption, quantified as 140 to 350 g/week for females and 210 to 420 g/week for males [3,13]. According to the consensus the cardiometabolic risk factors were as follows: (1) BMI > 25 kg/m^2^ or waist circumference >94 cm in male and >80 cm in female; (2) fasting glucose > 100 mg/dL or type 2 diabetes; (3) blood pressure > 130/85 mmHg or specific antihypertensive drug treatment; (4) triglycerides > 150 mg/dL or lipid-lowering treatment, (5) HDL-cholesterol ≤ 40 mg/dL in males and ≤50 mg/dL in females [3].

### 2.2. Study Procedures and Physical Examination

The diabetic patients included in the study were evaluated extensively within two visits, at the first evaluation and then after 12 months, noting the elements identified during the clinical examination, as well as their biological and imaging characteristics. A monitoring chart was created that included the following data: elements regarding the composition of the population in terms of age, sex, urban/rural environment; a complete anamnesis that included personal physiological and pathological antecedents, as well as heredocollateral ones, living and working conditions, smoking status, home treatment, medical history. A complete clinical examination (including calculation of body mass index (BMI), abdominal circumference (AC) and waist-to-height ratio (WtHr) was made. The cut-off values for normal weight (18.5–24.9 kg/m^2^), overweight (25–29.9 kg/m^2^), and obese (>30 kg/m^2^) people were used according to World Health Organization. As an additional measure of obesity, WtHr, which is calculated by dividing the waist circumference (in centimeters) by the height (in centimeters), was determined to be ≥0.50. Abdominal obesity was defined as a waist circumference of ≥80 cm for women and ≥94 cm for males [14].

### 2.3. Laboratory Tests and VCTE Examinations

Patients were biologically evaluated in a fasting state. The blood test included the determination of the blood count, evaluation of coagulation parameters, biochemical evaluation (liver, kidney function, blood sugar, glycated hemoglobin, insulinemia, iron, ferritin, cholesterol, triglycerides), immunological (to exclude hepatitis B and C viruses, autoimmune hepatitis), evaluation of tumor markers such as alpha-fetoprotein and carcinoembryonic antigen. The fibrosis-4 index (FIB-4) and NAFLD (NFS) score were also evaluated as serological indicators of liver fibrosis. For FIB-4 index, the cut-off values used to discriminate risk groups were: <1.30 for low risk, 1.30–2.6 for intermediate risk, and >2.67 for high-risk patients [15,16]. Age determines the cutoff values for the NFS, which is used for assessing the risk of advanced liver fibrosis. An NFS of less than −1.455 indicates a low risk of advanced fibrosis in people under 65, but an NFS of more than 0.676 indicates a high risk [17]. Standardized, validated questionnaires, such as the Alcohol Consumption Disorder Identification Test (AUDIT-C), were used to assess alcohol consumption to classify in current heavy drinking, active alcohol abuse, or alcohol dependence. The threshold for excessive alcohol consumption is 50 g per day for women and 60 g per day for males, according to recent scientific recommendations for patients with ALD [3,13].

Patients underwent abdominal ultrasound with evaluation of the characteristic elements of hepatic steatosis: hyperreflective liver, hepatorenal ultrasound contrast, hepatosplenic ultrasound contrast, vascular attenuation and blurring. A single operator with over 300 determinations in VCTE practice used the FibroScan^®^ 520 Compact model (Echosens, Paris, France) with the M- (normal) or XL- (obesity) probe to assess patients for liver fibrosis and steatosis [18]. Patients were in the supine posture with the right arm at maximal abduction following a minimum of four hours of fasting. This led to the expansion of the intercostal window for right lobe liver scanning. If ten acquisitions had an interquartile range divided by the median (IQR/M) of no more than 30%, the measurement was accepted as accurate [19]. At baseline and 12 months later, the LSM and CAP measurements were taken. The cut-off values for mild (S1), moderate (S2), and severe steatosis (S3) were, respectively, ≥274 dB/m, ≥290 dB/m, and ≥302 dB/m, based on CAP measurement. Regarding liver fibrosis, the cut-off of 5.6 kPa was for mild fibrosis (F1), 8 kPa for considerable fibrosis (F2), 9.6 kPa for advanced fibrosis (F3), and 13 kPa for cirrhosis (F4) [10].

### 2.4. Study Outcomes and Statistics

The main outcome was the comparative evaluation in terms of the evolution of liver fibrosis and steatosis in the two categories of patients, with MASLD and MetALD, as well as the comparative factors that improve or worsen underlying liver disease. Secondary outcomes included changes in body composition, liver function tests (LFTs), lipid metabolism, glucose metabolism indicators, and non-invasive testing for liver fibrosis (FIB-4 index, NFS-score, APRI score).

The statistical analysis was based on the use of both descriptive statistics and inferential methods, with the aim of synthesizing and interpreting the collected data in a rigorous and objective manner. The Shapiro–Wilk and Kolmogorov–Smirnov tests were used to determine whether the data distributions were normal. Depending on the test results, either the median (interquartile range) or the mean ± standard deviation was used to report the results. Independent samples were evaluated using the *t*-test and the Mann–Whitney U test, while differences between two dependent samples were examined using Student’s *t*-test and the Wilcoxon signed rank test. The Friedman test and repeated measures ANOVA were used to assess changes over time for related samples. The analyses were performed using SPSS 20.0 (IBM) and GraphPad Prism 7.0 (GraphPad Software). A two-sided *p*-value of less than 0.05 was used to assess statistical significance.

## 3. Results

### 3.1. Clinical and Biological Characteristics of the Studied Population

The baseline characteristics of our studied population are included in Table 1. We analyzed a total of 167 patients with MASLD and 119 patients with MetALD. From the MASLD cohort we found that 53.9% patients were males with a mean age of 57.84 ± 14.67 years. In the subgroup of patients with MetALD we found that 63% of them were males, with a mean age of 59.73 ± 13.29 years. Participants with MASLD had a higher mean BMI of 29.89 ± 4.29 kg/m^2^ with a prevalence of obesity of 64.1%, compared with MetALD subjects that had a BMI of 27.2 ± 3.89 kg/m^2^ and a prevalence of obesity of 37.8% with a significant statistic difference (*p* < 0.05). Also, the group of patients with MASLD had significantly higher values of WC and HOMA-IR levels compared with those with MetALD. By contrast, subjects with MetALD had higher values of fasting plasma glucose and HbA1c, but with no statistically significant difference (*p* > 0.05). Moreover, subjects with MetALD had significantly higher values of AST, ALT, GGT, ALP, bilirubin and AFP levels (*p* < 0.001) compared with those with MASLD, most likely due to alcohol consumption. Regarding the prevalence of hypertension and hyperlipidemia between MASLD and MetALD group there were no statistically significant differences, but patients with MASLD had an increased prevalence rate of metabolic syndrome (MS) of 68.3% compared with MetALD subjects that had a prevalence of MS of 55.5%. Additionally, we found there were significant statistical differences between those two groups regarding the medication for T2DM treatment (*p* < 0.001). Subjects with MASLD more commonly took SGLT-2 inhibitors (32.9%) and GLP-1 analogs (37.7%) and those with MetALD were most likely to consume Metformin (45.4%) for T2DM therapy.

### 3.2. Differences Between MASLD and MetALD Groups for Liver Steatosis and Fibrosis

The characteristics of liver steatosis and fibrosis for MASLD and MetALD subjects are analyzed in Table 2. Patients with MASLD present lower values of mean CAP of 313.32 ± 32.79 dB/m and had a prevalence of severe steatosis of 49.7% compared with subjects with MetALD, which had a mean CAP of 318.51 ± 35.97 dB/m and a prevalence of severe steatosis of 55.5%, but there are no significant differences between those two groups (*p* > 0.05). Regarding the levels of liver fibrosis, we found that participants with MetALD had higher values of LSM of 11.83 ± 6.27 kPa compared with MASLD subjects that had a mean LSM of 6.58 ± 2.27 kPa with a significant statistical difference (*p* < 0.001). Similarly, we found that subjects with MetALD had higher mean values of FIB-4 index (2.26 ± 1.75) and NFS-score (−0.97 ± 1.54) with a significant statistical difference (*p* < 0.001). Additionally, patients with MetALD had a significantly higher prevalence of advanced fibrosis of 17.6% and cirrhosis of 26.1% compared with MASLD participants, who had a prevalence of advanced fibrosis of 12.5% and a prevalence of cirrhosis of 4.2% (*p* < 0.001).

### 3.3. Differences Between Characteristics of Follow-Up Between MASLD and MetALD Patients

The clinical, biological, and imagistic characteristics of the participants at follow-up visit after 12 months are included in Table 3. In MASLD subjects, we found significantly higher levels of BMI with a mean value of 27.62 ± 3.83 kg/m^2^ and WC with a mean value of 97.75 ± 5.82 cm compared with MetALD participants, where the level of BMI was 26.4 ± 4.34 kg/m^2^ and the level of WC was 96.2 ± 6.51 cm (*p* value < 0.05). In contrast, patients with MetALD had increased values of HbA1c with a mean level of 6.41 ± 1.15%, compared with MASLD subjects that had a mean value of HbA1c at 6.16 ± 1.27% with a significant statistical difference (*p* value < 0.05). Moreover, in the MetALD group we found higher levels of fasting glucose with a mean value of 105.88 ± 36.11 mg/dL, but with no significant statistical difference (*p* value > 0.05). On the other hand, patients with MASLD had higher levels of triglycerides, HDL-cholesterol and HOMA-IR levels compared with MetALD subjects without statistical significance (*p* value > 0.05). The increased levels of cholesterol were obtained in MASLD group with a mean value of 198.94 ± 45.29 mg/dL, compared with the MetALD group where the mean value of cholesterol was 186.42 ± 60.95 mg/dL with a statistical difference (*p* value < 0.05). By contrast, subjects with MetALD had increased levels of INR, AST, ALT, GGT, ALP, bilirubin and AFP compared with MASLD participants with a significant statistical difference in all characteristics (*p* value < 0.001). Moreover, patients with MetALD had lower levels of albumin with a mean of 4.46 ± 0.67 g/dL and platelet count with a mean value of 215.42 ± 93.66 G/L, showing a significant difference from patients with MASLD (*p* value < 0.001).

Regarding the levels of liver steatosis, we found a slight increase in CAP at MetALD subjects with a mean value of 289.74 ± 36.70 dB/m compared with MASLD patients that had a mean CAP value of 288.23 ± 32.77 dB/m (*p* value > 0.05). On the other hand, patients with MASLD had lower levels of LSM with a mean value of 6.03 ± 2.54 kPa compared with MetALD subjects, who had a mean value of LSM at follow-up of 12.24 ± 8.66 kPa with a statistical difference between those two groups (*p* value < 0.001). Similarly, the levels of FIB-4 index and NFS-score were lower in the group of MASLD participants, with a mean value of 0.64 ± 0.49 and −2.46 ± 3.27, respectively, compared with MetALD subjects, with a statistically significant difference (*p* value < 0.05).

### 3.4. Evaluation of Liver Steatosis and Fibrosis at Follow-Up Visit

**Figure 2:**
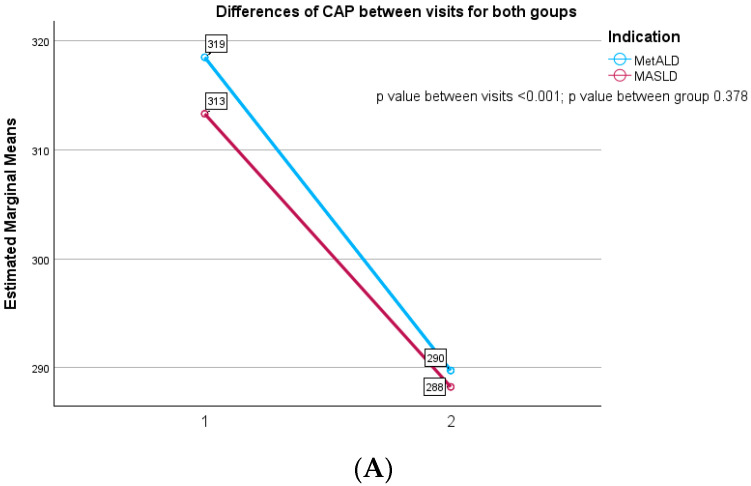

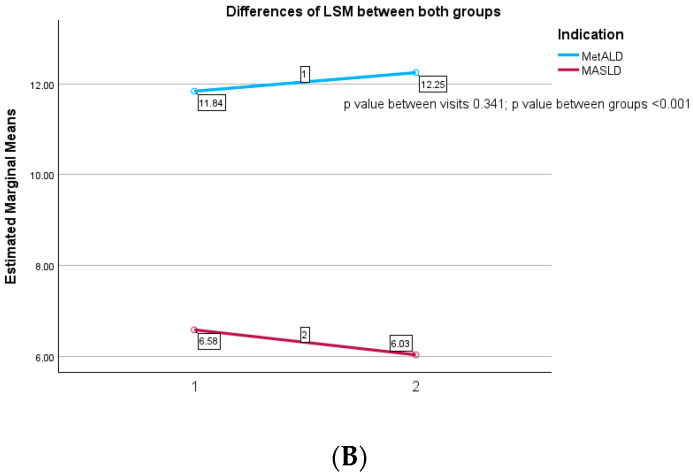
(**A**) Differences in CAP between visits for both groups. (**B**) Differences in LSM between visits for both groups.

According to Figure 2A, the CAP values decreased in MASLD patients from 313.32 ± 32.79 dB/m at the baseline to 288.23 ± 32.77 at 12 months follow-up visit with a significant statistical difference between evaluations (*p* value ≤ 0.001). Similarly, we observed a decrease in CAP values in MetALD subjects from 318.51 ± 35.97 dB/m to 289.74 ± 36.70 dB/m without any difference among these two groups of patients (*p* = 0.378). Concerning liver fibrosis evaluation, we analyzed in Figure 2B that MASLD subject presents a slight decrease in LSM values, from 6.58 ± 2.27 kPa to 6.03 ± 1.57 kPa at 12 months, without any statistical difference between the visits (*p* = 0.341). On the other hand, MetALD subjects had an increase of LSM values from 11.83 ± 6.27 kPa to 12.24 ± 8.66 kPa with a statistical difference between those two groups (*p* value ≤ 0.001).

**Figure 3:**
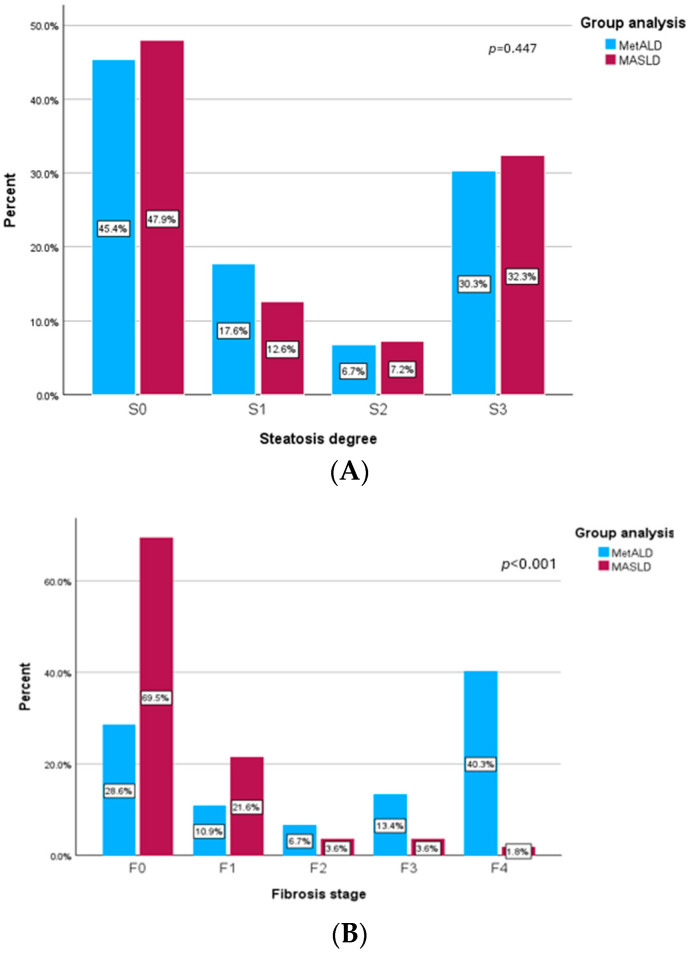
(**A**) Prevalence of steatosis degree at follow-up visit. (**B**) Prevalence of fibrosis stage at follow-up visit.

In Figure 3A, we analyzed the prevalence of steatosis degree at follow-up of 12 months for patients with MASLD and MetALD. We found that 47.9% of patients with MASLD had a decrease in CAP values below 274 dB/m, suggesting that they were in the S0 category of steatosis level. Moreover, approximately one third of the subjects with MASLD (32.3%) had severe steatosis (S3), 7.2% had moderate steatosis (S2) and 12.6% had mild steatosis (S1) after 12 months. Additionally, we found that 45.4% of MetALD participants had no steatosis at the follow-up visit, 17.6% had mild steatosis (S1), 6.7% had moderate steatosis (S2), and 30.3% of them had severe steatosis (S3). The results of liver steatosis levels were the same in the MASLD and MetALD groups with a *p* value of 0.447.

Regarding the levels of liver fibrosis at follow-up visit, we presented in Figure 3B the prevalence of fibrosis stage for both groups. MASLD subjects had lower values of LSM, and more than 90% of them were classified with no fibrosis (F0) or the mild stage (F1). More than that, only 3.6% of MASLD patients had significant fibrosis (F2) at the follow-up visit, 3.6% had advance fibrosis (F3), and 1.8% were classified with cirrhosis. On the other hand, MetALD patients had higher levels of liver fibrosis and only 39.5% of them had no fibrosis (F0) or mild fibrosis (F1). Additionally, in the MetALD group at the follow-up visit we found that 6.7% had significant fibrosis (F2), 13.4% and more than 50% of the patients had severe fibrosis (≥F3), with 40 of them having cirrhosis levels (LSM ≥ 13 kPa). Also, there were significant statistical differences between those two groups with a *p* value < 0.001.

### 3.5. Factors Associated with Liver Steatosis and Fibrosis Modifications on Follow-Up for Patients with MASLD or MetALD

In Table 4 we demonstrate a univariate linear regression to analyze the factors which are correlated with the decreased CAP values at the follow-up visit for MASDL and MetALD subjects, and only those factors which presented a significant correlation (*p* < 0.05) are included in the multivariate linear regression. For MASLD patients, we observed in univariate analysis that HOMA-IR at follow-up (β = 0.236, *p* = 0.019), BMI at follow-up (β = 0.244, *p* = 0.016) and WC at follow-up (β = 0.264, *p* = 0.009) are the only factors correlated with the reduced levels of CAP at 12 months visit. On the other hand, on the MetALD group the factors associated with the decreased values of CAP were HOMA-IR at follow-up (β = 0.188, *p* = 0.044) and cholesterol at follow-up (β = 0.227, *p* = 0.032). On the multivariate analysis, we found that the independent predictors for diminished values of CAP in MASLD groups were HOMA-IR at follow up (β = 0.178, *p* = 0.021), BMI at follow-up (β = 0.217, *p* = 0.017), WC at follow-up (β = 0.226, *p* = 0.011). Instead, for the MetALD patients the independent predictors correlated with decreased CAP values were HOMA-IR at follow-up (β = 0.195, *p* = 0.048), and cholesterol at follow-up (β = 0.188, *p* = 0.042).

In Table 5, we calculate the factors associated with the modification of LSM at follow-up for each group of patients. For the MASLD subjects, we find in univariate analysis that factors correlated with diminished LSM values were HOMA-IR at follow-up (β = 0.215, *p* = 0.002), fasting glucose at follow-up (β = 0.150, *p* = 0.026), ALT at follow-up (β = 0.204, *p* = 0.005), AST at follow-up (β = 0.126, *p* = 0.048), and FIB-4 index at follow-up (β = 0.380, *p* < 0.001). On the multivariate analysis, the independent predictors for diminished LSM values on MASLD subjects were HOMA-IR at follow-up (β = 0.257, *p* < 0.001), fasting glucose at follow-up (β = 0.137, *p* = 0.023), ALT at follow-up (β = 0.163, *p* = 0.023) and FIB-4 index at follow-up (β = 0.378, *p* < 0.001). By contrast, in MetALD group we observed that in univariate analysis the factors associated with increasing LSM values were ALT at follow-up (β = 0.137, *p* = 0.042), AST at follow-up (β = 0.166, *p* = 0.024), GGT at follow-up (β = 0.182, *p* = 0.02), and FIB-4 index (β = 0.647, *p* < 0.001). Additionally, on multivariate analysis we found that the factors which remained associated with increasing LSM values were AST at follow-up (β = 0.148, *p* = 0.036), GGT at follow-up (β = 0.158, *p* = 0.042), and FIB-4 index (β = 0.630, *p* < 0.001).

## 4. Discussion

Over time, obesity, T2DM, and other cardiometabolic risk factors have been widely accepted as associated factors with NAFLD. Although accumulating evidence has shown the importance of NAFLD, the use of stigmatizing terms to explain SLD has gradually necessitated the renaming of the term into a widely accepted nomenclature. Furthermore, because the pathophysiology of NAFLD, T2DM and ALD share similar pathophysiological processes, there is a need to clearly establish the contribution of alcohol in glycemic imbalance [20].

This study provides prospective evidence that diabetic patients with MetALD have significantly higher liver fibrosis burden and poorer glycemic control than those with MASLD alone, despite similar baseline steatosis severity. Alcohol appears to act synergistically with metabolic risk factors, accelerating fibrosis progression [21]. The World Health Organization recommends that neither men nor women drink more than 20 g/day of pure ethanol (two standard drinks). Alcohol consumption was the seventh leading risk factor for disability in 2016 and the leading cause of premature mortality and disability among young adults [22].

A prospective study found that alcohol consumption was directly proportional to the risk of developing MS [23]. Another study demonstrated the idea that alcohol consumption is associated with a higher incidence of MS in men, but no such association was found in women [24]. Alcohol consumption was not associated with MS in either sex in a longitudinal study of people aged 65 years or older. However, alcohol had a negative impact on blood glucose, waist circumference, and systolic blood pressure in men [25]. In contrast, Alkerwi et al. found that moderate alcohol consumption reduced the prevalence of MS in women [26].

In our study, we found that patients with MASLD had a higher mean BMI initially of 29.89 ± 4.29 kg/m^2^ with a prevalence of obesity of 64.1%, compared with MetALD subjects that had a BMI of 27.2 ± 3.89 kg/m^2^ and a prevalence of obesity of 37.8%. Also, the group of patients with MASLD had significantly higher values of WC and HOMA-IR levels compared with those with MetALD. By contrast, subjects with MetALD had higher values of fasting plasma glucose and HbA1c, highlighting the link between alcohol consumption and insufficient glycemic control.

Hong-ye Peng et al. analyzed 1862 eligible individuals diagnosed with MASLD and Met-ALD. They found that an elevated risk of hypertension, T2DM, and hyperlipidemia is linked to MASLD. For the MASLD patients, there was a significant and independent correlation between the degree of hepatic steatosis and the probability of T2DM. A higher risk of hypertension was linked to MetALD [27]. Regarding our research, during the follow-up visit, we observed that in the MetALD group we found higher levels of fasting glucose with a mean value of 105.88 ± 36.11 mg/dL. On the other hand, patients with MASLD had higher levels of triglycerides, HDL-cholesterol and HOMA-IR levels.

Oh et al. investigated the effectiveness of non-invasive diagnostics performed in diagnosing patients with MASLD and MetALD. In this study, 7918 Korean health check-up subjects in total who had both ultrasound and magnetic resonance elastography evaluated to diagnose hepatic steatosis were included. Although not statistically significant, the LSM was greater in the MetALD group than in the MASLD group (4.6 vs. 4.5 kPa). In contrast, the MetALD group’s CAP was noticeably lower (275 vs. 285 dB/m). Age, sex, BMI, and the prevalence of T2DM, hypertension, or dyslipidemia did not significantly differ between the two groups [28].

Our study demonstrated that patients diagnosed with MASLD initially exhibited lower mean CAP values (313.32 ± 32.79 dB/m) and a prevalence of severe steatosis of 49.7%, in comparison to individuals with MetALD, whose mean CAP was 318.51 ± 35.97 dB/m and prevalence of severe steatosis was 55.5%. Evaluation of liver fibrosis levels indicated that participants with MetALD had higher mean LSM values (11.83 ± 6.27 kPa) versus MASLD subjects (mean LSM 6.58 ± 2.27 kPa). At the follow-up visit, assessment of liver steatosis showed a slight increase in CAP among MetALD subjects (mean 289.74 ± 36.70 dB/m), whereas MASLD patients presented a mean CAP value of 288.23 ± 32.77 dB/m. Conversely, MASLD patients displayed lower LSM levels at follow-up (mean 6.03 ± 2.54 kPa) compared to MetALD subjects (mean LSM 12.24 ± 8.66 kPa).

Another study, which used the National Health and Nutrition Examination Survey (NHANES) data set from 2017 to 2020, evaluated the presence of MASLD, MetALD and ALD, and inclusion criteria were represented by adult participants with complete TE examinations. Thus, 9698 patients were included. The authors investigated which risk factors—such as age, gender, ethnicity, diabetes, hypertension, obesity, increased WC, dyslipidemia, and moderate alcohol use—are linked to clinically severe liver fibrosis in patients with MASLD and MetALD. Male gender and a higher ethnicity-adjusted WC were linked to clinically severe fibrosis in a multivariate logistic regression. Additionally, advanced fibrosis was linked to T2DM and male gender. When compared to patients with MASLD, moderate alcohol use was not related to an increased risk of severe or advanced fibrosis [29].

In a study that analyzed the interrelationship between MS and the evolution of liver disease, it was concluded that MS increases the risk of liver complications, regardless of the level of alcohol consumption. Metabolic components appear to modify the correlation between alcohol consumption and the risk of liver disease. The group was divided into distinct categories according to the frequency of alcohol consumption (either occasional or regular/daily) and the presence of the MS component (either absent or present), establishing that the risk of liver dysfunction increased in tandem with the frequency of alcohol consumption [30].

In our cohort, for MASLD patients, we observed in the multivariate analysis that the independent predictors for diminished values of CAP in MASLD groups were HOMA-IR at follow up, BMI at follow-up, and WC at follow-up. For the MetALD patients the independent predictors correlated with decreased CAP values were HOMA-IR at follow-up, and cholesterol at follow-up.

Our findings align with prior studies showing greater fibrosis in MetALD, but the magnitude of difference in our cohort, especially in cirrhosis prevalence, is notable. The improvement in CAP in both groups suggests potential reversibility of steatosis with management, but persistent or worsening fibrosis in MetALD highlights alcohol’s disproportionate role in structural liver damage.

Strengths include the prospective design, standardized TE measurements, and inclusion of a relatively large diabetic cohort. Limitations include reliance on self-reported alcohol intake without biomarkers, hospital-based recruitment (possible selection bias), absence of histologic confirmation, and a relatively short follow-up for chronic liver disease outcomes. Additionally, CAP and LSM cut-offs are not validated specifically for MetALD, which may affect classification. Clinically, these results underscore that even moderate alcohol intake in diabetic patients warrants careful monitoring and lifestyle counseling, as its impact on fibrosis progression is substantial.

## 5. Conclusions

This study highlights that patients with MetALD had a significantly higher risk of severe progression of chronic liver disease due to more advanced fibrosis levels compared to those with MASLD. The economic, social and health system burden of liver disease among diabetic patients warrants screening for MASLD and MetALD. In summary, moderate alcohol use combined with glycemic imbalance raises the risk of liver disease progression more than poor glycemic control alone. Thus, it is necessary to develop public health policies that highlight the negative role of alcohol consumption in any quantity in diabetic patients.

## Figures and Tables

**Table 1:** Baseline characteristics of participants with MASLD and MetALD.

	MASLD Patients (*n* = 167)	MetALD Patients (*n* = 119)	*p*-Value
Gender, *n* (%)			0.12
Female	77 (46.1%)	44 (37%)	
Male	90 (53.9%)	75 (63%)	
Age (years)	57.84 ± 14.67	59.73 ± 13.29	0.26
BMI (kg/m^2^)	29.89 ± 4.29	27.2 ± 3.89	<0.001
BMI classification, *n* (%)			<0.001
Normal Weight	31 (18.6%)	49 (41.2%)	
Overweight	29 (17.4%)	25 (21%)	
Obesity	107 (64.1%)	45 (37.8%)	
Smoking, *n* (%)	42 (25.1%)	66 (55.5%)	<0.001
WC (cm)	101.15 ± 9.98	95.28 ± 12.5	<0.001
Hb1Ac (%)	6.65 ± 1.24	6.88 ± 1.15	0.113
HOMA-IR	2.09 ± 0.11	2.06 ± 0.1	0.01
TG (mg/dL)	174.45 ± 58.54	172.22 ± 56.68	0.74
Cholesterol (mg/dL)	226.93 ± 35.09	225.48 ± 37.26	0.73
HDL-C (mg/dL)	45.35 ± 10.72	45.03 ± 9.45	0.78
Fasting glucose (mg/dL)	127.81 ± 45.31	129.33 ± 33.07	0.75
Platelet count (G/L)	269.8 ± 58.76	213.43 ± 85.14	<0.001
Ferritin (mg/dL)	126.06 ± 64.33	147.78 ± 110.51	0.037
INR	1.03 ± 0.19	1.11 ± 0.2	0.003
ALT (IU/L)	32.39 ± 15.49	58.96 ± 44.55	<0.001
AST (IU/L)	26.02 ± 10.7	54.12 ± 47.41	<0.001
GGT (IU/L)	43.32 ± 34.89	135.78 ± 103.22	<0.001
ALP (IU/L)	85.35 ± 30.77	103.68 ± 56.62	<0.001
Bilirubin (mg/dL)	0.72 ± 0.38	0.96 ± 0.59	<0.001
Albumin (g/dL)	4.55 ± 0.45	4.47 ± 0.55	0.21
AFP (ng/mL)	3.21 ± 1.35	4.99 ± 3.31	<0.001
Creatinine (mg/dL)	0.83 ± 0.17	0.85 ± 0.21	0.28
Presence of hypertension, n(%)	92 (55.1%)	72 (60.5%)	0.36
Presence of hyperlipidemia, *n* (%)	105 (62.9%)	70 (58.8%)	0.49
Presence of MS, *n* (%)	114 (68.3%)	66 (55.5%)	0.02
Medication, *n* (%)			<0.001
*Diet*	5 (3%)	6 (5%)	
*Metformin*	39 (23.4%)	54 (45.4%)	
*SGLT2-inhibitors*	55 (32.9%)	25 (21%)	
*GLP1-analogs*	63 (37.7%)	34 (28.6%)	
*Insulin therapy*	5 (3%)	0 (0%)	

Abbreviations: BMI, body mass index; INR, international normalized ratio; ALT, alanine aminotransferase; AST, aspartate aminotransferase; GGT, gamma-glutamyl transpeptidase; ALP, alkaline phosphatase.

**Table 2:** Differences between MASLD and MetALD groups for liver steatosis and fibrosis.

	MASLD Patients (*n* = 167)	MetALD Patients (*n* = 119)	*p*-Value
*CAP (dB/m)*	313.32 ± 32.79	318.51 ± 35.97	0.2
*Steatosis degree, n (%)*			0.41
*Mild steatosis (S1)*	51 (30.5%)	33 (27.7%)	
*Moderate steatosis (S2)*	33 (19.8%)	20 (16.8%)	
*Severe steatosis (S3)*	83 (49.7%)	66 (55.5%)	
*LSM (kPa)*	6.58 ± 2.27	11.83 ± 6.27	<0.001
*Fibrosis stages, n (%)*			<0.001
*No fibrosis (F0)*	51 (30.5%)	23 (19.3%)	
*Mild fibrosis (F1)*	55 (32.9%)	34 (28.6%)	
*Moderate fibrosis (F2)*	33 (19.7%)	10 (8.4%)	
*Advanced fibrosis (F3)*	21 (12.57%)	21 (17.6%)	
*Cirrhosis (F4)*	7 (4.19%)	31 (26.1%)	
*FIB-4 index*	1.02 ± 0.4	2.26 ± 1.75	<0.001
*NFS-score*	−1.78 ± 1.25	−0.97 ± 1.54	<0.001

Abbreviations: MASLD, metabolic dysfunction-associated steatotic liver disease; MetALD, metabolic dysfunction-associated steatotic liver disease with moderate alcohol intake; CAP, controlled attenuation parameter; LSM, liver stiffness measurements; FIB-4, fibrosis-4 index; NFS-score, NAFLD, Non-Alcoholic Fatty Liver Disease Fibrosis score; No fibrosis (F0); S1-Mild steatosis; S2-Moderate steatosis; S3-Severe steatosis; F1-Mild fibrosis; F2-Moderate fibrosis; F3-Advanced fibrosis; F4-Cirrhosis.

**Table 3:** Differences between characteristics of follow-up between MASLD and MetALD patients.

	MASLD Patients (*n* = 167)	MetALD Patients (*n* = 119)	*p*-Value	Standardized Difference	95% CI
BMI (kg/m^2^)	27.62 ± 3.83	26.4 ± 4.34	0.006	0.48	[−2.17; −0.26]
WC (cm)	97.75 ± 5.82	96.2 ± 6.51	0.018	0.73	[−2.99; 0.10]
Hb1Ac (%)	6.16 ± 1.27	6.41 ± 1.15	0.048	0.14	[−0.04; 0.53]
HOMA-IR	2.13 ± 0.10	2.11 ± 0.10	0.06	0.01	[−0.04; 0.005]
TG (mg/dL)	138.7 ± 69.61	134.68 ± 72.56	0.318	8.49	[−20.75; 12.71]
Cholesterol (mg/dL)	198.94 ± 45.29	186.42 ± 60.95	0.024	6.28	[−24.88; −0.15]
HDL-c (mg/dL)	51.89 ± 14.82	53.65 ± 15.6	0.166	1.81	[−1.81; 5.34]
Fasting glucose (mg/dL)	100.45 ± 31.89	105.88 ± 36.11	0.090	4.04	[−2.53; 13.38]
Platelet count (G/L)	280.79 ± 58.76	215.42 ± 93.66	<0.001	9.02	[−83.14; −46.6]
INR	0.84 ± 0.19	1.11 ± 0.31	<0.001	0.03	[0.21; 0.32]
ALT (IU/L)	35.16 ± 15.29	55.52 ± 37.46	<0.001	4.43	[14.03; 26.7]
AST (IU/L)	17.87 ± 6.24	48.42 ± 36.82	<0.001	30.54	[21.82; 39.26]
GGT (IU/L)	35.90 ± 26.71	130.69 ± 98.44	<0.001	20.36	[60.58; 96.99]
ALP (IU/L)	73.46 ± 38.88	113.01 ± 74.99	<0.001	19.79	[26.13; 52.94]
Bilirubin (mg/dL)	0.7 ± 0.33	1.10 ± 0.7	<0.001	0.39	[0.27; 0.51]
Albumin (g/dL)	4.72 ± 0.5	4.46 ± 0.67	<0.001	0.26	[−0.39; −0.12]
AFP (ng/mL)	3.29 ± 1.26	4.85 ± 2.78	<0.001	1.56	[0.8; 2.04]
*CAP (dB/m)*	288.23 ± 32.77	289.74 ± 36.70	0.35	4.13	[−6.62; 9.64]
*NFS-score*	−2.46 ± 3.27	−1.17 ± 3.81	0.03	1.29	[−0.05; 2.63]
*FIB-4 index*	0.64 ± 0.49	2.33 ± 1.61	<0.001	1.68	[1.42; 1.94]
*LSM (kPa)*	6.03 ± 1.57	12.24 ± 8.66	<0.001	1.44	[9.8; 12.47]

Abbreviations: MASLD, metabolic dysfunction-associated steatotic liver disease; MetALD, metabolic dysfunction-associated steatotic liver disease with moderate alcohol intake; BMI, body mass index; INR, international normalized ratio; ALT, alanine aminotransferase; AST, aspartate aminotransferase; GGT, gamma-glutamyl transpeptidase; ALP, alkaline phosphatase; HDL-c, high-density lipoprotein cholesterol; CAP, controlled attenuation parameter; LSM, liver stiffness measurements; FIB-4, fibrosis-4 index; NFS-score, NAFLD, Non-Alcoholic Fatty Liver Disease Fibrosis score.

**Table 4:** Univariate and multivariate linear regression analysis of factors associated with decreased CAP values in MASLD and MetALD subjects at follow-up.

	MASLD Group				MetALD Group			
	Univariate		Multivariate		Univariate		Multivariate	
Parameter	Β	*p*-value	Β	*p*-value	Β	*p*-value	β	*p*-value
HOMA-IR at follow-up	0.236	0.019	0.178	0.021	0.188	0.044	0.195	0.048
Cholesterol at follow-up	-	-	-	-	0.227	0.032	0.188	0.042
BMI at follow-up	0.244	0.016	0.217	0.017	-	-	-	-
WC at follow-up	0.264	0.009	0.226	0.011	-	-	-	-

Abbreviations: MASLD, metabolic dysfunction-associated steatotic liver disease; MetALD, metabolic dysfunction-associated steatotic liver disease with moderate alcohol intake; HOMA-IR, Homeostasis Model Assessment of Insulin Resistance; BMI, body mass index; WC, waist circumference.

**Table 5:** Univariate and multivariate linear regression analysis of factors associated with modification of LSM values in MASLD and MetALD subjects at follow-up.

	MASLD Group				MetALD Group			
	Univariate		Multivariate		Univariate		Multivariate	
Parameter	Β	*p*-value	β	*p*-value	β	*p*-value	β	*p*-value
HOMA-IR at follow-up	0.215	0.002	0.257	<0.001	-	-	-	-
Fasting glucose at follow-up	0.150	0.026	0.137	0.045	-	-	-	-
ALT at follow up	0.204	0.005	0.163	0.023	0.137	0.042	-	-
AST at follow up	0.126	0.048	-	-	0.166	0.024	0.148	0.036
GGT at follow up	-	-	-	-	0.182	0.02	0.158	0.042
FIB-4 index at follow up	0.380	<0.001	0.378	<0.001	0.647	<0.001	0.630	<0.001

Abbreviations: MASLD, metabolic dysfunction-associated steatotic liver disease; MetALD, metabolic dysfunction-associated steatotic liver disease with moderate alcohol intake; HOMA-IR, Homeostasis Model Assessment of Insulin Resistance; ALT, alanine aminotransferase; AST, aspartate aminotransferase; GGT, gamma-glutamyl transpeptidase; FIB-4, fibrosis-4 index.

## Data Availability

The data presented in this study are available on request from the corresponding author. The data are not publicly available because they are the property of the Institute of Gastroenterology and Hepatology, Iasi, Romania.
